# Pathogen-specific alterations in intestinal microbiota precede urinary tract infections in preterm infants: a longitudinal case-control study

**DOI:** 10.1080/19490976.2024.2333413

**Published:** 2024-04-01

**Authors:** Luyang Hong, Yihuang Huang, Junyan Han, Shujuan Li, Lan Zhang, Qi Zhou, Xincheng Cao, Weiyin Yu, Xinhui Guo, Yi Yang, Yufeng Zhou, Weili Yan, Shangyu Hong, Siyuan Jiang, Yun Cao

**Affiliations:** a Department of Neonatology, Children’s Hospital of Fudan University, Shanghai, China; b NHC Key Laboratory of Neonatal Diseases, Children’s Hospital of Fudan University, Shanghai, China; c National Children’s Medical Center, Department of Clinical Epidemiology of Children’s Hospital of Fudan University, Shanghai, China; d State Key Laboratory of Genetic Engineering, School of Life Sciences, Fudan University, Shanghai, China

**Keywords:** Preterm infants, urinary tract infection, microbiome, virulence factor, calprotectin

## Abstract

Urinary tract infections (UTIs) are among the most common late-onset infections in preterm infants, characterized by nonspecific symptoms and a pathogenic spectrum that diverges from that of term infants and older children, which present unique diagnostic and therapeutic challenges. Existing data on the role of gut microbiota in UTI pathogenesis in this demographic are limited. This study aims to investigate alterations in gut microbiota and fecal calprotectin levels and their association with the development of UTIs in hospitalized preterm infants. A longitudinal case-control study was conducted involving preterm infants admitted between January 2018 and October 2020. Fecal samples were collected weekly and analyzed for microbial profiles and calprotectin levels. Propensity score matching, accounting for key perinatal factors including age and antibiotic use, was utilized to match samples from UTI-diagnosed infants to those from non-UTI counterparts. Among the 151 preterm infants studied, 53 were diagnosed with a UTI, predominantly caused by Enterobacteriaceae (79.3%) and Enterococcaceae (19.0%). Infants with UTIs showed a significantly higher abundance of these families compared to non-UTI infants, for both Gram-negative and positive pathogens, respectively. Notably, there was a significant pre-UTI increase in the abundance of pathogen-specific taxa in infants later diagnosed with UTIs, offering high predictive value for early detection. Shotgun metagenomic sequencing further confirmed the dominance of specific pathogenic species pre-UTI and revealed altered virulence factor profiles associated with Klebsiella aerogenes and Escherichia coli infections. Additionally, a decline in fecal calprotectin levels was observed preceding UTI onset, particularly in cases involving Enterobacteriaceae. The observed pathogen-specific alterations in the gut microbiota preceding UTI onset offer novel insight into the UTI pathogenesis and promising early biomarkers for UTIs in preterm infants, potentially enhancing the timely management of this common infection. However, further validation in larger cohorts is essential to confirm these findings.

## Introduction

Urinary tract infections (UTIs) are among the most common bacterial infections in preterm infants,^1–3^ characterized by nonspecific symptoms^4,5^ and a pathogenic spectrum distinct from that of term infants and older children.^1,4,6^ The complexity of diagnosing and treating UTIs in this vulnerable population is compounded by their immature immune system and the unique challenges posed by their developmental stage. Furthermore, clinical decompensation secondary to UTIs in preterm infants may result in the need for interruption of enteral feeding, increased respiratory support, and prolonged hospital stay, and late-onset sepsis due to urinary tract infection in very preterm neonates is not uncommon.^2,5^ Therefore, investigation into the pathogenesis and potential biomarkers of UTIs in preterm infants is urgently warranted for early diagnosis and optimal treatment.

The pathogenesis of UTI in preterm infants is not well understood. Previous studies indicated that blood-borne infections are the main pathogenesis of UTIs in premature infants.^2,5,7^ However, recent evidence suggested a link between gut microbial composition and the susceptibility to UTIs in various populations, reinforcing the concept of an “ascending” pathogenesis where gut pathogens migrate to the urinary tract.^8–11^ Preterm infants face several environmental and physiologic challenges that may often predispose them to dysbiosis, including the unique environment in the neonatal intensive care units (NICUs), invasive devices and broad-spectrum antibiotic use, and immature intestinal development.^12–15^ As a result, preterm infants own a unique, dynamic, and complex gut microbiota, dominated by opportunistic pathogens including *Klebsiella* and *E.coli*,^13,14,16^ which may serve as a reservoir for early-life infections. In particular, the pathogen-specific alterations in microbiota profiles before sepsis onset have been discovered.^17,18^ To the best of our knowledge, data on the relationship between gut microbiota and UTI in preterm neonates remains scarce. Longitudinal investigation into the gut microbiota changes in UTI progression may offer a promising avenue for early detection and prevention strategies.

On the other hand, the immaturity of the gastrointestinal system and unfavorable microbiota-host interactions may also contribute to the susceptibility of infection in preterm infants.^12^ Recent studies have shown that calprotectin, a 36-kDa protein secreted by local immune cells, plays key roles in the regulation of immunity development and the promotion of beneficial bacterial colonization during infancy.^19^ Therefore, fecal calprotectin (FC) may be a potential indicator for intestinal immunity, and its interactions with the intestinal flora in preterm infants after birth are worth exploring, especially in the context of infectious diseases.^20^

In light of the above, this longitudinal case-control study aims to combine information on the dynamics of gut microbiota and calprotectin levels in the progression of UTI in preterm infants to delve into UTI pathogenesis.

## Materials and methods

### Study design and participants

The study was conducted in the NICU of Children’s Hospital of Fudan University from January 2018 to October 2020. In the prospective cohort, all infants with gestational age <32 weeks or with birth weight < 1500 grams and admitted to NICU within 24 hours of life during the study period were enrolled and followed prospectively by the research team. The exclusion criteria include major congenital anomalies, inborn error of metabolism, sepsis (excluded unless the diagnosis of sepsis is within 72 hours of UTI onset), confirmed NEC, and hospitalization of fewer than seven days. Infants with no available samples were excluded from the study. The Ethics Committee of the Children’s Hospital of Fudan University approved this study (No. 2018(149)). Oral informed consent was obtained from the parents of the participants.

### Definitions

Blood culture and urine culture were performed simultaneously on or after postnatal day 7 when infection was suspected. All samples used for urine culture were obtained by trained doctors through bladder catheterization after local sterile disinfection. UTI was defined as isolation of a single pathogenic organism with a colony count of ≥ 50,000 CFU/mL from the urine collected.^21^ Urine cultures with isolation of more than one organism were considered a potential contaminant, and a new urine culture was performed immediately afterward. Multiple positive urine cultures for the same organism within 7 days were considered collectively as a single UTI. Urinalysis was not routinely performed and therefore was not available for analysis. Infants with UTI were categorized into Gram-positive or Gram-negative pathogen groups according to the Gram-staining classification of the corresponding UTI pathogen. In terms of infants with recurrent UTI onsets, each infant was classified into Gram-positive or Gram-negative pathogen groups according to their first UTI pathogen. NEC was defined as neonates with stage ≥II NEC according to the Bell criteria.^22^ Sepsis includes both culture-proven and culture-negative sepsis. Culture-proven sepsis was defined by a positive blood culture. Culture-negative sepsis was diagnosed when all the following criteria were fulfilled: 1) infection-related clinical manifestations; 2) abnormal white blood cell count, C-reactive protein level, or procalcitonin level; 3) antibiotics used or intended for ≥5 days; 4) negative blood culture; 5) no evidence of concurrent focal infection.^23,24^ Early-onset sepsis was defined as sepsis diagnosed within 72 h of postnatal age.^25^ Infants without UTI, necrotizing enterocolitis, or sepsis were defined as controls. Gestational age was determined using the hierarchy of best obstetric estimates or gestational age estimation using the Ballard Score.^26^ Small for gestational age was defined as birth weight < 10th percentile for the gestational age according to the Chinese neonatal birth weight values.^27^

### Fecal sample collection, preparation, and FC measurement

Fecal samples were collected weekly from admission to discharge from all enrolled infants. Fecal samples were immediately stored at 0°C and transferred to −80°C within 1 h for long-term storage until thawed for further analysis. Meconium was defined as the stool passed within 72 h after birth.

FC levels were determined using a monoclonal ELISA kit (EK-CAL, Bühlmann, Schö- nenbuch, Switzerland), according to the manufacturer’s instructions. FC levels were expressed as micrograms per gram (µg/g) of feces. FC levels below the detection threshold (30 µg/g) were replaced with the minimum value (30 µg/g). Samples with FC values larger than the detection range (1600 µg/g) were further retested after dilution to obtain the exact values.

### Stool 16S rRNA gene sequencing and microbial analysis

The 16S rRNA sequencing and raw data preprocessing were processed according to the standard protocols provided by Majorbio Bio-Pharm Technology Co. Ltd. (Shanghai, China), as previously described.^28^ In brief, microbial genomic DNA was isolated from each fecal specimen using the E.Z.N.A. Soil DNA Kit (Omega Bio-Tek, Norcross, GA, U.S.) according to the manufacturer’s instructions. The V3-V4 hypervariable region of the bacterial 16S rRNA gene was sequenced using universal primers pairs 338F (5’-ACTCCTACGGGAGGCAGCAG-3’) and 806 R (5’-GGACTACHVGG GTWTCTAAT-3’) using a thermo- cycler PCR system (GeneAmp 9700, Applied Biosystems, USA). Equimolar amounts of purified amplicons were pooled and paired ended sequenced (2 × 300) on an Illumina MiSeq platform (Illumina, San Diego, CA, USA) according to the standard protocols. Chimera was detected and removed by UCHIME Algorithm. Taxonomic identity was assigned to the resulting Operational Taxonomic Units (OTUs; 97% similarity) by alignment to the Silva (SSU123) 16S rRNA database using a confidence threshold of 70%. Samples with a total read of less than 20,000 were removed (*n* = 1). The median and interquartile range (IQR) of the read distribution was 28,532 and 16 reads, respectively. Sequences that were present only once in each sample were further filtered. Predominance of bacteria at different taxa (phylum, family, or genus level) was defined by the taxa that having the highest prevalence in the corresponding sample. BugBase (https://bugbase.cs.umn.edu/) was used for the evaluation of the abundance of bacteria of different Gram-staining (Gram_Negative and Gram_Positive) in each sample. Taxa bar plots of microbial composition and principal coordinate analysis (PCoA) were generated using the ‘phyloseq’^29^ (ver. 1.30.0) package. Permutational multivariate analysis of variance (PERMANOVA) was performed to determine factors that explained variance in bacterial community compositions of samples using the “Adonis” function in the “vegan” package (ver. 2.5–6) based on 2,000 permutations. Differences in the relative abundance of the microbial features were determined by linear discriminant analysis (LDA) effect size (LEfSe).^30^ The SplinectomeR^31^ R package and its permuspliner functions were used to assess significant differences in the longitudinal abundances of bacterial taxa between UTI and non-UTI groups using standard 999 permutations.

### Metagenomic sequencing

Total genomic DNA of the human feces was extracted using the E.Z.N.A.® Soil DNA Kit. After extraction, DNA concentration was measured by TBS-380, DNA purity by NanoDrop2000, and DNA integrity by 1% agarose gel electrophoresis. The genomic DNA was fragmented by Covaris M220 (Gene Company Limited, Hong Kong, China), and screened for fragments of about 400bp. PE libraries were constructed using the NEXTFLEX Rapid DNA-Seq kit (Bioo Scientific, Austin, USA). After amplification by bridge PCR, macro-genome sequencing was performed using the Illumina NovaSeq/Hiseq Xten (Illumina, San Diego, USA) sequencing platform at Majorbio Bio-Pharm Technology Co. Ltd. Quality control of the raw data was performed by Fastq software, and BWA was used to compare the reads to the host DNA reads and contaminating reads with high comparative similarity were removed. The optimized sequences were spliced using splicing software based on the succinct de Bruijn graphs principle. Contigs ≥ 300 bp were selected from the spliced results as the final assembly results. The ORF prediction of the assembled contigs was performed using MetaGene, and then the predicted gene sequences were clustered using CD-HIT software to construct a non-redundant gene set. Finally, using SOAPaligner software, high-quality reads of each sample were compared with the non-redundant gene set (95% identity) to count the abundance of the genes in the corresponding samples. The amino acid sequences of the non-redundant gene set were aligned with the Integrated genomes from the gut and other environments dataset^32^ using BLASTP to obtain annotations at Species level.

### Abundance of virulence factors

The abundance of antibiotic resistance genes and virulence factors was detected and quantified in each sample. For this, we used DIAMOND^33^ to align metagenomic sequence data against protein reference databases. First, protein sequences of the core and predicted virulence factor dataset were downloaded from the Virulence Factors Database (VFDB, http://www.mgc.ac.cn/VFs/).^34^ Second, for each sample, metagenomics sequence reads were aligned to both reference protein databases using DIAMOND. Alignment was only considered valid if both paired-reads aligned to the same protein sequence. In the cases of multiple matches, only the best match was kept. This was specified by using the DIAMOND alignment option “−k 1”. Proteins abundances were quantified as counts per million (CPM): calculated by the raw valid counts (number of valid alignments) divided by the library sizes and multiplied by one million. After excluding genes present in less than 5% of the samples, the abundances of 853 virulence factor proteins were analyzed.

### Propensity matching of infants’ fecal samples

To assess the differences in UTI progression between UTI group and controls, we employed propensity score matching (PSM) in R to identify control samples with similar demographics and clinical characteristics to those of UTI infants at different time points relative to the day of UTI diagnosis. Using PSM, samples of non-UTI neonates were matched to UTI infant samples with a ratio of 3:1. The continuous variables included in the models were postnatal age, gestational age, birth weight, Apgar scores at 5 minutes, and accumulated days of antibiotic use or probiotic use prior to the days of sample collection. The binary variables included gender, delivery mode, and feeding mode. The matching was achieved using the optimal matching algorithm without replacement.

### Statistical analysis and data integration

Statistical analyses and graphical outputs were performed using R version 4.0 and RStudio unless stated otherwise. For all analyses, the normality of data was tested with the Shapiro-Wilk test using “shapiro.test” and “normality” from the “dlookr” package. Categorical variables were compared by using Pearson chi-squared tests or Fisher’s exact tests when appropriate; Continuous data were compared using the Student’s t-test or Mann – Whitney U test for two groups when appropriate, or using the one-way analysis of variance or Kruskal-Wallis rank sum test for three groups when appropriate. Parametric and non-parametric pairwise comparisons were made using the R package rstatix. Graphs were created using the R packages ggplot2 and ggpubr. Correlations between different indexes were explored using Spearman’s methods. *P*-values were adjusted for false discovery rate (FDR) using the Benjamini-Hochberg method. The significance level was determined by a *P*-value <.05 or an FDR *P*-value <.1.

## Results

### Clinical and demographical characteristics

**Figure 1.**
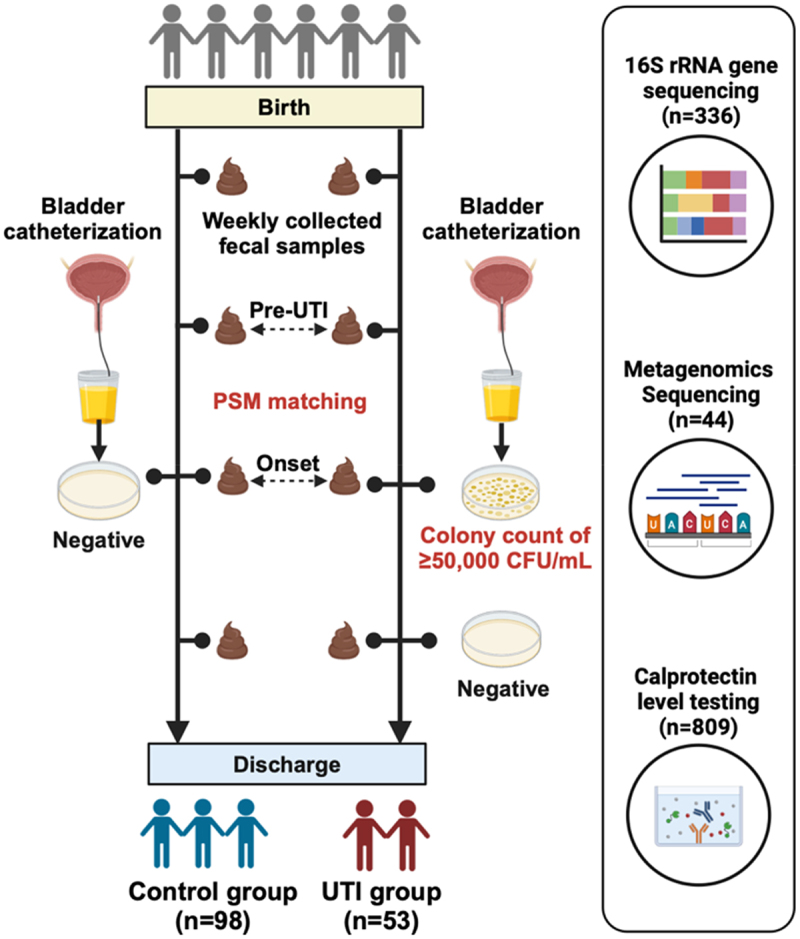
Study design and sample analysis.

**Figure 2.**
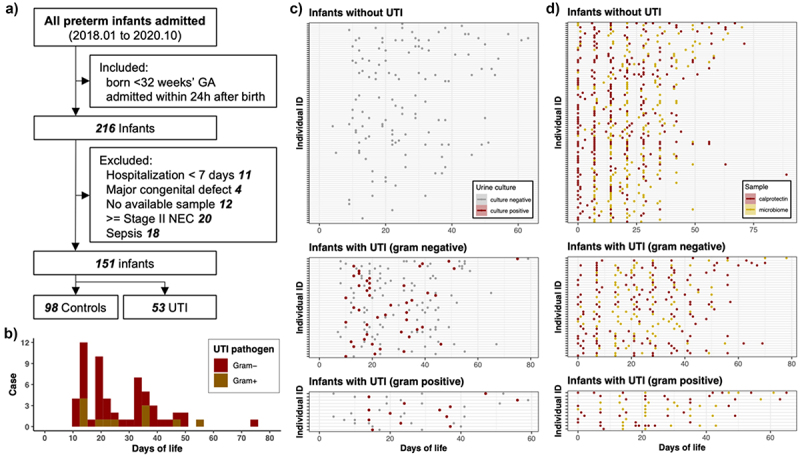
Study design and sample characteristics in UTI infants and controls.

Between January 2018 and October 2020, 151 preterm infants were included in the study, of whom 53 were diagnosed with UTI and 98 infants without sepsis or NEC served as controls (Figures 1 & 2a). The demographic and clinical variables between groups and metadata of all infants were elucidated in Table 1. Compared to the controls, infants with UTIs had lower gestational age (median [IQR]: 30.2 [1.6] vs. 29.5 [1.8] weeks, *p* value = .012) and birth weight (median [IQR]: 1330 [175.7] vs. 1273.9 [187.9] grams, *p* value = .07), a higher proportion of males (n/N [%]: 37/98 [37.8%] vs. 37/53 [69.8%], *p* value < .001), and a longer of days of antibiotic use (median [IQR]: 10.4 [8.0] vs. 22.4 days [9.5], *p* value < .001) and length of hospitalization (median [IQR]: 47.8 [13.8] vs. 57.2 [13.6] days, *p* value < .001).

(a) Flow chart of study design. (b) Distribution of UTI onset according to the gram staining of the corresponding UTI pathogens, respectively. (c) Time points all urine cultures in enrolled infants during hospitalization. The points in red and gray color represent urine culture with positive and negative results, respectively. The X-axis and Y-axis represent different days of postnatal age and different infants, respectively. (d) Distribution of collected fecal samples of all enrolled infants during hospitalization. The X-axis and Y-axis represent different days of postnatal age and different infants, respectively. Fecal samples were all analyzed for calprotectin level, and those further analyzed for 16S rRNA sequencing were displayed in yellow color.

**Table 1.** Demographics and clinical variables of UTI and non-UTI group.

Variable	All included infants			Samples after PSM matching		
	non-UTI infant	UTI infant	P-value	non-UTI sample	UTI sample	P-value
	(*N* = 98)	(*N* = 53)		(*n* = 135)	(*n* = 45)	
**Infant characteristics**						
Gestational age (weeks), mean (SD)	**30.24 (1.62)**	**29.49 (1.84)**	.**012**	29.94 (1.68)	29.50 (1.77)	.137
Birth Weight (g), mean (SD)	1329.95 (176.00)	1273.87 (187.86)	.07	1305.59 (174.50)	1262.11 (185.08)	.156
Male, n/N (%)	**37 (37.8)**	**37 (69.8)**	**<.001**	70 (51.9)	30 (66.7)	.119
Small for gestational age, n/N (%)	9 (9.2)	6 (11.3)	.893	9 (6.7)	5 (11.1)	.52
Apgar score at 1 minute, mean (SD)	7.81 (1.62)	7.47 (2.01)	.267	7.57 (1.53)	7.20 (2.32)	.223
Apgar score at 5 minutes, mean (SD)	8.77 (1.01)	8.51 (1.17)	.163	8.56 (1.03)	8.24 (1.26)	.093
**Maternal characteristics**						
Multiple pregnancy, n/N (%)	40 (40.8)	17 (32.1)	.378	58 (43.0)	14 (31.1)	.219
Vaginal delivery, n/N (%)	29 (29.6)	19 (35.8)	.545	52 (38.5)	16 (35.6)	.859
PPROM, n/N (%)	20 (20.4)	16 (30.2)	.252	33 (24.4)	14 (31.1)	.425
Use of prenatal antibiotics, n/N (%)	17 (17.3)	12 (22.6)	.567	21 (15.6)	10 (22.2)	.347
Gestational diabetes mellitus, n/N (%)	20 (20.4)	9 (17.0)	.769	32 (23.7)	7 (15.6)	1
gestational hypertension, n/N (%)	9 (9.2)	4 (7.5)	.969	11 (8.1)	3 (6.7)	.57
**Treatment**						
Exclusive breastmilk feeding, n/N (%)	73 (74.5)	40 (75.5)	1	36 (26.7)	12 (26.7)	1
Antibiotics use (days)*, mean (SD)	**10.40 (7.96)**	**22.43 (9.47)**	**<.001**	6.39 (5.75)	8.02 (6.70)	.116
Probiotics use (days)*, mean (SD)	6.10 (11.08)	5.51 (9.07)	.739	0.99 (5.05)	0.93 (4.46)	.951
Hospital stay/Days of life (days)**, mean (SD)	**47.78 (13.81)**	**57.19 (13.56)**	**<.001**	22.81 (14.53)	23.47 (13.93)	.793

*For all included infants, antibiotics use and probiotics use in both groups were calculated as the accumulated days of antibiotics and probiotics use throughout hospitalization. For samples after PSM matching, antibiotics use and probiotics use in both groups were calculated as the accumulated days of antibiotic use or probiotic use prior to the days of sample collection.

**Days of total hospital stay for all included infants. Days of life for samples after PSM matching.

Abbreviation: UTI, urinary tract infection. PPROM, prolonged premature rupture of membrane.

In all enrolled infants, 312 urine cultures were performed during their stay in the hospital, and 58 onsets of UTI from 53 UTI infants were cataloged (Table 2 & Figure 2b, c). The onset of UTI occurred predominantly in between 10 and 20 days and between 40 to 50 postnatal days (Figure 2b). The UTI pathogens were predominantly Gram-negative bacteria of the *Enterobacteriaceae* family (79.3%, including *Klebsiella spp*. [*n* = 31], *Enterobacter cloacae* [*n* = 9], and Escherichia *coli* [*n* = 4], etc.), followed by Gram-positive bacteria *Enterococcus faecalis* (*n* = 10, 17.2%). Mean (IQR) postnatal age at UTI onset was 26.7 (20.8) days. Five infants experienced two UTI onsets during their stay in NICU (Table S1), and the mean (IQR) days between the two UTI onset was 25 (7) days. Among these infants, three had a recurrence of UTI due to the original pathogen. Notably, within 72 hours of UTI diagnosis, 5 infants had blood cultures Indicating bacteremia, with isolates including *Staphylococcus capitis* (three cases) and *Staphylococcus epidermidis* (two cases). There was no overlap of species in urine and blood specimens.

**Table 2.** Distribution and characteristics of UTI pathogens.

UTI pathogen	Gram	Family	Number of Cases	Age at onset (median)	Age of onset (IQR)	Age of onset (max)	Age of onset (min)
E.faecalis	Gram+	Enterococcaceae	10	30	16.25	56	14
E.faecium	Gram+	Enterococcaceae	1	14	0	14	14
S.aureus	Gram+	Staphylococcaceae	1	15	0	15	15
K.pneumoniae	Gram-	Enterobacteriaceae	18	29	15.75	44	13
K.aerogenes	Gram-	Enterobacteriaceae	9	32	23	49	10
E.cloacae	Gram-	Enterobacteriaceae	9	15	3	33	10
E.coli	Gram-	Enterobacteriaceae	4	29.5	22.25	75	13
K.oxytoca	Gram-	Enterobacteriaceae	4	19.5	9.25	46	12
C.koseri	Gram-	Enterobacteriaceae	1	17	0	17	17
S.marcescens	Gram-	Enterobacteriaceae	1	51	0	51	51

### Perturbed gut microbiota development in UTI infants was characterized by elevated pathogen-specific taxa abundance prior to infection onset

**Figure 3.**
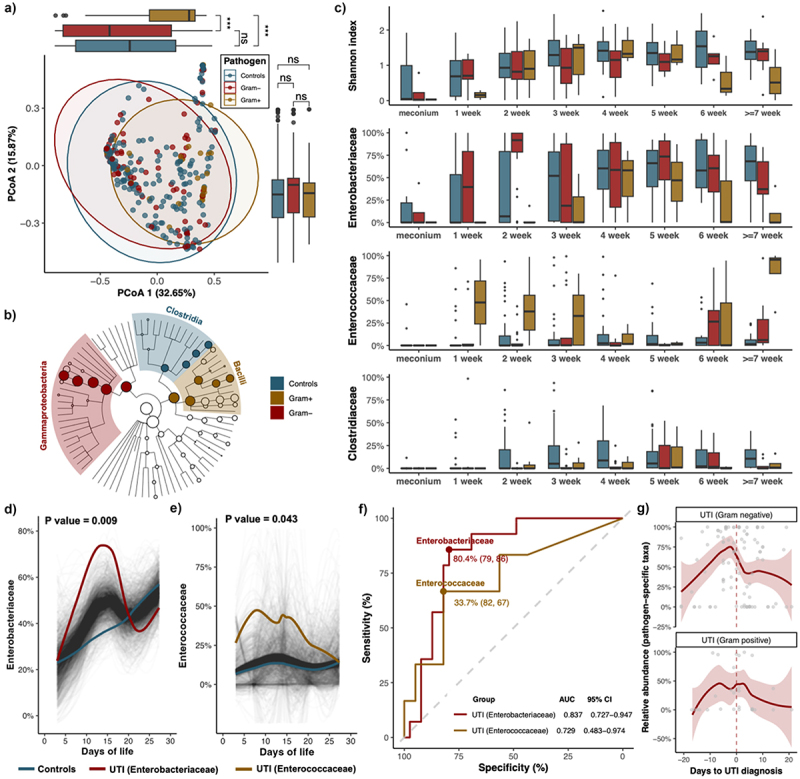
Disrupted gut microbiome dynamics and increased abundance of pathogen-associated gut bacteria in UTI infants.

A total of 809 and 336 samples were analyzed for calprotectin levels and 16S rRNA gene sequencing, respectively (Figure 2d & Table S2). The principal coordinate analysis (PCoA) plot showed that the overall microbial distribution of infants with Gram-positive UTI pathogens was different from that of infants with Gram-negative UTI pathogens and controls (PERMANOVA test: R^2^ = 1.1%, *p* value = .003), especially on the PCoA1 axis (Figure 3a). The abundance of *Clostridia*, mostly *Clostridium_sensu_stricto_1*, was significantly enriched in the controls (LEfSe: adjusted *p* value < 0.1), while *Gammaproteobacteria* and *Bacilli* were enriched in Gram-negative UTI and Gram-positive UTI group, respectively (Figure 3b & Figure S1A-B). From the perspective of bacterial alpha diversity, the Shannon index of the Gram-positive UTI group was lower than that of the other groups in the first week and after the sixth week of postnatal age (Figure 3c). Notably, UTI infants with different pathogens exhibited distinct microbial development trajectories (Figure 3c & Figure S1C-D). Specifically, a rapid surge in *Enterobacteriaceae* abundance was identified at two weeks of postnatal age in infants with Gram-negative UTI pathogens. Conversely, those with Gram-positive UTI pathogens manifested a persistent elevation in *Enterococcaceae* abundance throughout their hospital stay, particularly in the first three weeks of life. Next, we investigated the longitudinal changes in pathogen-specific bacterial abundance between samples of infants with and without UTI within the first month of age using the SplinectomeR (Figure 3d, e). We observed significant longitudinal differences in the abundance of *Enterobacteriaceae* (*p* value = .009) and *Enterococcaceae* (*p* value = .043) between controls and UTI infants with the corresponding pathogens. Subsequently, ROC analysis was performed in fecal samples collected between 10 to 15 postnatal days to examine the effects of fecal microbial abundance in distinguishing pre-UTI from non-UTI samples. With a cutoff abundance of 80.4% for *Enterobacteriaceae* and 33.7% for *Enterococcaceae*, the relative abundance of these two taxa distinguished subsequent UTI onset due to the corresponding pathogen from non-UTI infants with a sensitivity of 79% and 86% and a specificity of 82% and 67%, respectively (Figure 3f).

(a) The overall microbial profiles in infants with UTI (Gram-positive and Gram-negative pathogens) and controls using principal-coordinate analysis (PCoA) based on the Bray-Curtis dissimilarity at the genus level (n = 336). Percentages shown are the percentage of the variation explained by the corresponding principal coordinate, and the first two were displayed. Dots and boxplots in blue, red, and yellow color represent controls, UTI patients with Gram-negative pathogens, and UTI patients with Gram-positive pathogens, respectively. (b) Taxonomic cladogram depicting taxonomic association between microbiome communities among groups. Each node represents a specific taxonomic type. Nodes in blue, yellow, and red denote the taxonomic types with more abundance in controls, Gram-positive UTI, and Gram-negative UTI groups, respectively (LEfSe: adjusted *p* value < 0.1 & LDA > 3). Significantly altered taxa at the class level are highlighted and labeled accordingly. (c) Distribution of Shannon index and the relative abundance of *Enterobacteriaceae* and *Enterococcaceae* according to postnatal age in each group. (d-e) Longitudinal changes in *Enterobacteriaceae* (d) and *Enterococcaceae* (e) abundance between controls and UTI infants with the corresponding pathogens, using SplinectomeR with permuted spline test (permutations = 999). (f) ROC curves to analyze the performance of fecal microbial abundance as a biomarker to distinguish pre-UTI and non-UTI in samples collected between 10 to 15 days of age. Lines and dots in red and yellow color represent the AUC curve and the optimal cut-point of the relative abundance of *Enterobacteriaceae* (39 controls and 14 cases) and *Enterococcaceae* (39 controls and 6 cases) to distinguish UTI and non-UTI with the corresponding pathogen, respectively. AUC, area under curve; CI, confidence intervals. (g) Change of relative abundance of pathogen-specific taxa at family level according to the time of UTI onset. Smoothed lines result from LOESS (locally estimated scatterplot smoothing) and indicate a longitudinal change of all data.

We next assessed the longitudinal shift in gut microbiome according to UTI progression and found significant UTI pathogen-associated alterations (Figure 3g & Figure S2). Specifically, within one week preceding UTI onset, there was a surge in the relative abundance of *Enterobacteriaceae* and *Enterococcaceae* in infants with Gram-negative and Gram-positive UTI pathogens, respectively (Figure 3g & Figure S2a). At the genus level, this trend of increased abundance of pathogen-specific taxa in UTI infants prior to UTI onset was also observed (Figure S2B-C).

**Figure 4.**
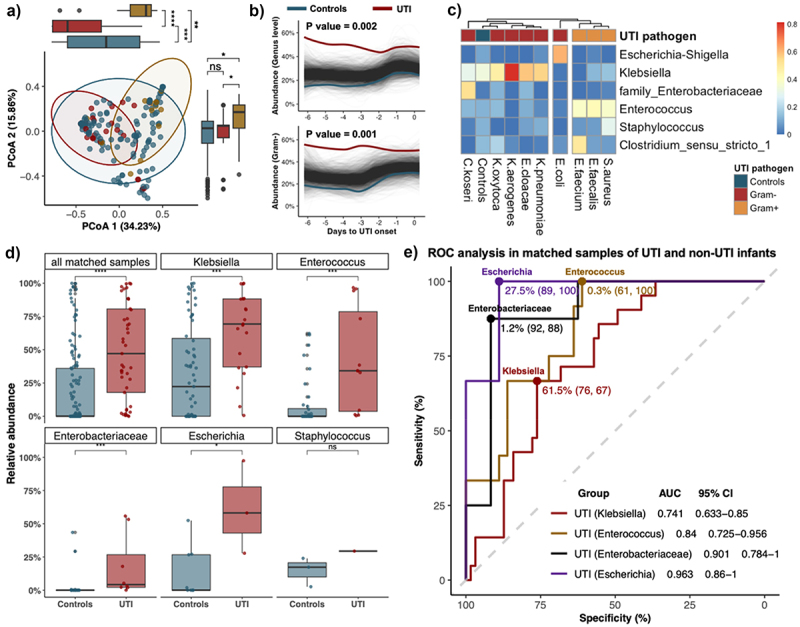
Distinct pathogen-specific gut microbiome profiles in UTI infants prior to infection onset.

To probe into the gut microbiome profiles preceding UTI, we further analyzed stool samples collected from one week before to 24 hours post UTI onset in UTI infants and matched samples using propensity scores. Specifically, each pre-UTI samples were matched with three samples from the controls (*n* = 180), accounting for major clinical variables including postnatal age, accumulated days of antibiotic use or probiotic use, gestational age, birth weight, Apgar scores at 5 minutes, gender, delivery mode, and feeding mode. There were no significant differences in major demographic and clinical variables between matched samples of infants with and without UTI (Table 1). Among all the matched samples, PCoA plots revealed distinct gut microbiota profiles between controls and infants with different pathogens (PERMANOVA test: R^2^ = 4.8%, *p* value < .001; Figure 4a). Notably, 84.4% (27/32) of pre-UTI fecal samples in infants with Gram-negative UTIs were dominated by Gram-negative bacteria, and 61.5% (8/13) of infants with Gram-positive UTIs were dominated by Gram-positive bacteria (Figure S3A). Prior to UTI onset, we also observed significant longitudinal differences in the abundance of UTI-specific taxa at the genus level between matched samples of UTI and non-UTI infants (*p* value = .002), especially in those with Gram-negative UTI pathogens (*p* value = .001; Figure 4b). Heatmap revealed that the intestinal flora of Gram-negative and Gram-positive UTI cases clustered separately, and the gut microbial profiles of most UTI infants were dominated by the corresponding pathogen-specific genera (Figure 4c). In parallel, compared to the matched controls, the abundance of pathogen-specific genera was significantly higher in pre-UTI samples in most UTI cases, including those with UTIs due to *Klebsiella*, *Enterococcus*, unclassified *Enterobacteriaceae* (including *E. cloacae* and *C. koseri*), *Escherichia*, and *Staphylococcus* (Figure 4d & Figure S3). The pathogen-specific genera still dominated the gut of UTI infants in most of the pre-UTI samples when examined the pathogens at the species level, and their abundance was significantly elevated when compared to the matched controls (Figure S3C-D). The effect of the microbial abundance of pathogen-specific genera in distinguishing UTIs from matched controls was further examined by ROC analysis. With a cutoff value of 27.5%, the relative abundance of *Escherichia* distinguished UTI infants with the corresponding pathogens from matched samples with a sensitivity of 89% and a specificity of 100% (AUC = 0.963). For *Enterobacteriaceae* (including *E. cloacae* and *C. koseri*), *Enterococcu*s, and *Klebsiella*, the cutoff abundance were 1.2%, 0.3%, and 61.5%, with sensitivities of 92%, 61%, and 67%, and specificities of 88%, 100%, and 76%, respectively (AUC between 0.741 to 0.901, Figure 4e).

(a) The microbial profiles of pre-UTI samples in infants with UTI (Gram-positive and Gram-negative pathogens) and matched samples of controls using principal-coordinate analysis (PCoA) based on the Bray-Curtis dissimilarity at the genus level (*n* = 180). Percentages shown are the percentage of the variation explained by the corresponding principal coordinate, and the first two were displayed. (b) Longitudinal changes in pathogen-specific abundance at genus level between matched samples of all UTI infants and non-UTI infants (up) and between matched samples of UTI infants with Gram-negative pathogens and non-UTI infants (down), using SplinectomeR with permuted spline test (permutations = 999). (c) The heatmap revealed the mean abundance of the major genus of matched samples of controls and UTI patients with different pathogens. (d) Boxplot revealed the difference in microbial abundance at the genus level of matched samples between UTI infants and non-UTI infants, respectively. *Enterobacteriaceae* refers to the abundance of unclassified *Enterobacteriaceae* in 16S rRNA gene sequencing profiles. UTI infants in this category include those with UTI due to *E. cloacae* and *C. koseri*. (e) ROC curves to analyze the performance of fecal microbial abundance as a biomarker to distinguish UTI from non-UTI in matched samples of all infants. Lines and dots in red and yellow color represent the AUC curve and the optimal cut-point of the relative abundance of genus to distinguish UTI from non-UTI with the corresponding pathogen, respectively. AUC, area under curve; CI, confidence intervals.

### Species-level microbiome variations and altered virulence profiles in preterm infants with UTI

**Figure 5.**
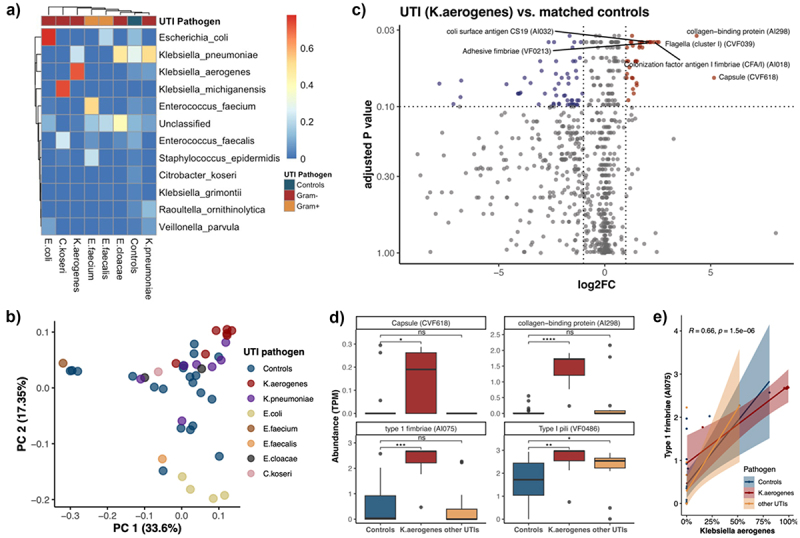
Pathogen-specific gut microbiome profiles at species level and altered virulence profiles UTI infants prior to infection onset.

Shotgun metagenomic sequencing was performed in a subset of fecal samples from UTI infants (*n* = 23) and controls (*n* = 21) (Table S5). Heatmap revealed the dominance of pathogen-specific species in most of UTI cases, especially that of *Escherichia coli*, *Klebsiella aerogenes, Klebsiella pneumoniae*, and *Enterococcus faecalis* (Figure 5a).

(a) Heatmap revealed the abundance of major species of metagenomic samples of UTI patients and controls. (b) The overall VFDB profiles in infants with UTI and controls using principal-coordinate analysis (PCoA) based on the Bray-Curtis dissimilarity at the genus level. Percentages shown are the percentage of the variation explained by the corresponding principal coordinate, and the first two were displayed (*n* = 44). (c) Volcano plot revealed the differential abundance of virulence factors of *Klebsiella aerogenes* UTI patients and matched controls (*n* = 21). (d) Boxplot revealed differences in virulence factors between *Klebsiella aerogenes* UTI infants, UTI infants with other pathogens, and controls, respectively. (e) Correlations between the relative abundance of *Klebsiella aerogenes* and Type 1 fimbriae (AI075) in all samples using Spearman’s method. Boxplots, points, and lines in red, yellow, and blue color were *Klebsiella aerogenes* UTI infants, UTI infants with other pathogens, and controls, respectively.

We further analyzed virulence factors including those that act by increasing bacterial adhesion to the intestinal mucosa, evading the immune system, or suppressing the host immune response.^35^ The homology between our metagenomic reads and the protein sequences from the Virulence Factor Database (VFDB; *n* = 853) was assessed. PCA revealed different virulence factor profiles in UTI cases, especially that of *Escherichia coli* and *Klebsiella aerogenes* (Figure 5b). Specifically, in infants with *Escherichia coli* infection, the abundance of 40 virulence factors was increased compared with matched controls (FDR <0.1; Table S8), with the most pronounced increases including colonization factor antigen III (CFA/III) and Colicin-like Usp, which have been identified to play vital roles in *E. coli* colonization and promotion of ascending UTIs, respectively (Figure S4).^36–38^ In infants with *Klebsiella aerogenes* infection, the abundance of 94 virulence factors was increased (Figure 5c & Table S8), notably, capsules and those associated with the bacterial adhesive capacity, including fimbriae, pili, and collagen-binding protein (Figure 5d). Among all samples, the abundance of type I fimbriae (AI075) was significantly correlated with the relative abundance of *Klebsiella aerogenes* (Spearman’s rho = 0.66; *p* value = 1.5 × 10^−6^; Figure 5e).

### Decreased fecal calprotectin levels before UTI onset and its correlation with pathogen abundance

**Figure 6.**
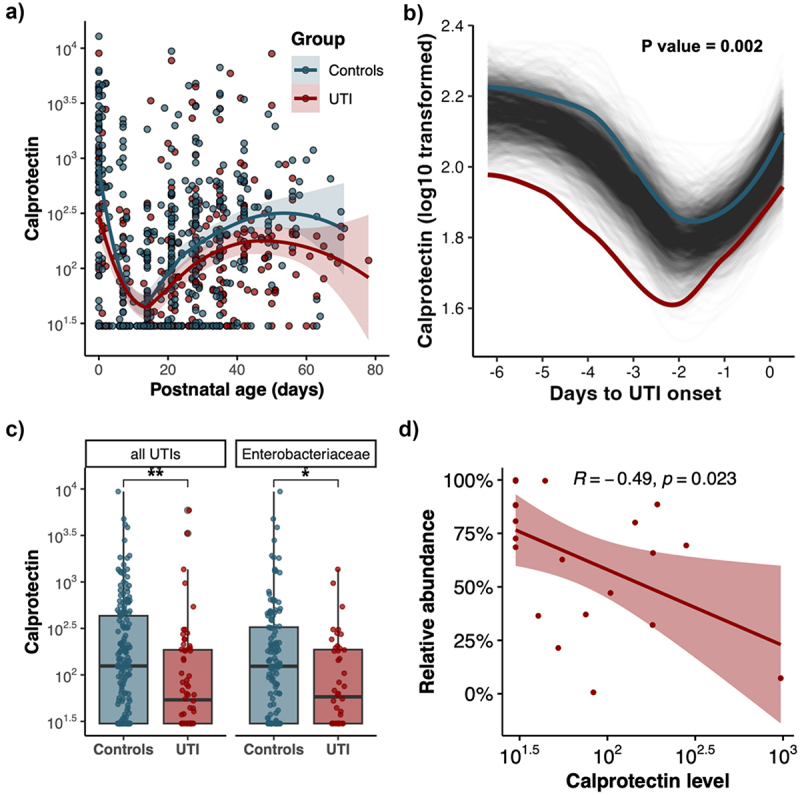
Altered fecal calprotectin level and its association with pathogen abundance prior to infection onset in UTI infants.

To examine the involvement of gut immunity and its correlation with the microbiome in the pathogenesis of UTI, the longitudinal changes of fecal calprotectin levels in preterm infants with and without UTI were examined (*n* = 809). Compared with the controls, the postnatal FC levels in infants with UTI were observed to be decreased throughout hospitalization (Figure 6a). We further examined the FC levels in relation to UTI onset with a focus on those prior to UTI onset (*n* = 256) (Table S4). Prior to UTI onset, we also observed significant longitudinal differences in the fecal calprotectin level between matched samples of UTI and non-UTI infants (Figure 6b). Compared to the matched controls, the level of calprotectin was significantly decreased in stools prior to UTI onset, chiefly in UTIs with *Enterobacteriaceae* infections (Figure 6c & Figure S5). In addition, we also observed a significantly negative association between fecal calprotectin level and *Klebsiella* abundance in pre-UTI fecal samples in UTI patients with *Klebsiella spp*. infections (Spearman’s rho = −0.49, *p* value = .021) (Figure 6d). No significant association was observed between fecal calprotectin level and relative abundance of the pathogen-specific genus in pre-UTI fecal samples in other UTI patients.

(a) Longitudinal changes of fecal calprotectin (FC) levels according to postnatal age in infants with or without UTI, respectively (*n* = 809). (b) Longitudinal changes in log10 transformed calprotectin levels in matched samples of all UTI infants and non-UTI infants, using SplinectomeR with permuted spline test (permutations = 999). (c) Boxplot revealed the difference in FC levels of matched samples between controls and all UTI patients (*n* = 256) and Enterobacteriaceae-related UTI infants (*n* = 176), respectively. (d) Correlations between FC levels and the relative abundance of Klebsiella in samples of Klebsiella UTI infants using Spearman’s method (*n* = 21).

## Discussions

In this longitudinal case-control study, we illustrated profound pre-UTI alterations in gut microbial profiles and calprotectin levels which were closely associated with different UTI pathogens. These findings pinpointed the possible involvement of gut dysbiosis and unfavorable host-microbial interactions in the pathogenesis of UTIs in preterm infants.

UTI is one of the most common infections in preterm infants in NICUs, presenting a high incidence around the world.^2,3,39–41^ In our center, urine cultures were routinely performed through catheterization when infants present with nonspecific manifestations associated with infection, especially among those at more than one week of age, and a relatively high incidence of UTI was yielded. Similar to other preterm infant populations,^2–4,40,41^ the pathogen spectrum of UTIs in this study was dominated by the *Enterobacteriaceae* family, including pathogenic organisms *Klebsiella* and *Escherichia*. To date, there are no guidelines or consensus published regarding the management of UTIs in preterm infants around the world.^6,42,43^ Therefore, it is necessary to investigate the pathogenesis of UTI for optimal management in preterm infants.

Historically, an overarching perspective concerning UTIs in preterm infants emphasized the bloodstream as the origin of UTI pathogens.^2,3,7^ However, in light of the increasing emphasis on the gut as a repository of UTI pathogens in other populations^8–11,35^ and the unfavorable intestinal microbial features of preterm infants,^13,14,16^ we investigated the relationship between gut microbiota and UTIs in preterm infants. Through longitudinal observation, our results revealed that UTI infants with different pathogens exhibited distinct microbial development trajectory. Notably, we captured a notable surge of UTI pathogen-related taxa in the gut before UTI onset, confirming the role of gut flora in UTI susceptibility and its potential as a diagnostic and therapeutic target in preterm infants. Of particular note, the abundance of *Enterobacteriaceae* and *Enterococcaceae*, two most common pathogens in UTI infections, grew rapidly around two weeks of age in the gut in UTI infants with the corresponding pathogens. In this period, the abundance of pathogen-specific taxa showed relatively good sensitivity and specificity in distinguishing UTI infants from controls in ROC analyses. Since the high prevalence of UTIs also occurs around two weeks after birth, monitoring alterations in gut flora in preterm infants during this period may be beneficial for the early detection and prevention of UTIs. Furthermore, we also found a rapid surge of pathogen-specific taxa in pre-UTI samples collected within one week before the UTI onset at the genus and species level. Our findings suggest a potential ‘ascending’ pathogenesis, where pathogens may migrate from the stool to the urinary tract via the skin or diaper. This perspective differs from the traditionally discussed gut-organ axes in the literature and points toward an environmentally acquired UTI. Such an understanding emphasizes the importance of environmental and hygiene factors in the prevention and management of UTIs in preterm infants. These results also suggested the great potential of longitudinal monitoring of gut microbial composition in the early diagnosis of UTI, prediction of pathogens, as well as the optimal use of antibiotics in preterm infants. However, we acknowledge the complexity of clinical and demographic variables in preterm infants, which include significant inter- and intra-individual variations. It is important to note that infants diagnosed with UTI differed in some key clinical covariates from those who did not develop UTI, potentially influencing the gut microbiome and its implications for UTI pathogenesis. While our analysis employed propensity score matching to mitigate the effects of these variables, we cannot exclude the possibility of residual confounding. On the other hand, in cases of UTIs due to pathogens other than *Enterobacteriaceae* and *Enterococcaceae*, which were rare or absent in this study, the relation between gut bacteria abundance and UTI-pathogen could not be determined. Further research is needed to validate these findings in larger and more diverse cohorts.

Furthermore, our data also revealed altered virulence factor profiles of pre-UTI samples, particularly *Escherichia coli* and *Klebsiella*, which may be associated with their ability to establish infections.^35^ The virulence factors that stood out in our results in *Escherichia coli* include colonization factor antigen III (CFA/III) and Colicin-like Usp, which are key factors for bacterial colonization of the intestine^37^ and endothelial cell damage^36^ as well as an increased incidence of UTIs.^38^ The markedly elevated virulence factors in UTI of *Klebsiella* are attributed to adhesion factors, including fimbriae and pili, which may enhance the ability of pathogens to colonize and ascend. However, it is noteworthy that the abundance of *Escherichia coli* and *Klebsiella* in UTI infants with corresponding pathogen was also significantly higher compared to controls, so it is not possible to determine whether the elevation in virulence factors was due to their own changes or the elevated bacterial abundance. In addition, the virulence factor was not investigated in samples after UTI onset, which prevented us from investigating the effect of therapeutic factors including antibiotic use and its association with recurrent UTIs. In future studies, there is a need for direct analysis of pathogen virulence factors in urine cultures of UTI infants to clarify, on the one hand, the changes in the virulence factors of UTI pathogens as compared to controls and their influence by antibiotic use, and, on the other hand, the correlation between changes in virulence factors of UTI pathogens and that of the corresponding bacteria in the gut.

Another pivotal observation was the notable decrease in fecal calprotectin levels during the periods preceding UTI onset, which plays an important role in early immune development and gut microbiome colonization.^19^ Its reduced levels, particularly in the context of rising pathogenic bacteria, may reflect a compromised or suppressed local immune response. In addition, a negative correlation between calprotectin level and *Klebsiella* abundance was observed in pre-UTI stools, consistent with a previous study reporting a negative correlation between fecal calprotectin levels and *Enterobacteriaceae* abundance.^19^ These observations, coupled with our microbial findings, suggest that UTIs in preterm infants may be a combined outcome of altered gut microbiota and possibly altered gut immunity. This confluence of factors could create an environment conducive to pathogen colonization and subsequent migration, leading to UTIs. However, the information provided by only calprotectin levels as an indicator of intestinal immunity was also limited, and our results provided a preliminary suggestion of a possibility. More indicators and rigorous studies will be needed in the future to determine the role of intestinal immune-microbiome interactions in the pathogenesis of UTI in preterm infants.

## Conclusions

Profound pathogen-specific preclinical alterations underline that gut microbiota and gut immunity marker may be involved in the pathogenesis of UTI in preterm infants. Longitudinal monitoring of gut microbiota and calprotectin levels may contribute to the early prevention and optimal management of this vulnerable group. More in-depth microbial research is warranted.

## Supplementary Material

Supplemental Figures_revised.docx

Supplemental Tables_revised.xlsx

## Data Availability

The 16S rRNA sequencing files and metagenomics files were submitted to the National Center for Biotechnology Information Sequence Read Archive (https://www.ncbi.nlm.nih.gov/sra, accessed on 1 October 2023) and are available with BioProject accession number PRJNA1079890. The other supplemantary files have been made available on Figshare.com via the DOI: 10.6084/m9.figshare.24603270.
