# Supplementary material for: Pathogen-specific alterations in intestinal microbiota precede urinary tract infections in preterm infants: a longitudinal case-control study
Source: Gut Microbes. 2024 Apr 1;16(1):2333413. doi: 10.1080/19490976.2024.2333413 (PMC10986765; doi:10.1080/19490976.2024.2333413)
Supplement: Supplemental Figures_revised.docx [file KGMI_A_2333413_SM2131.docx]

**Figures**

**Figure S1.** Disrupted microbial development trajectory in stools of UTI infants.

**Figure S2.** Increased abundance of pathogen-associated taxa in stools of UTI infants.

**Figure S3.** Distinct pathogen-specific gut microbiome profiles in UTI infants prior to infection onset.

**Figure S4.** Volcano plot revealed the differential abundance of virulence factors of Escherichia coli UTI patients and matched controls.

**Figure S5.** Change of calprotectin level in matched samples of UTI infants and controls.

**Figure S1.** **Disrupted microbial development trajectory in stools of UTI infants**.


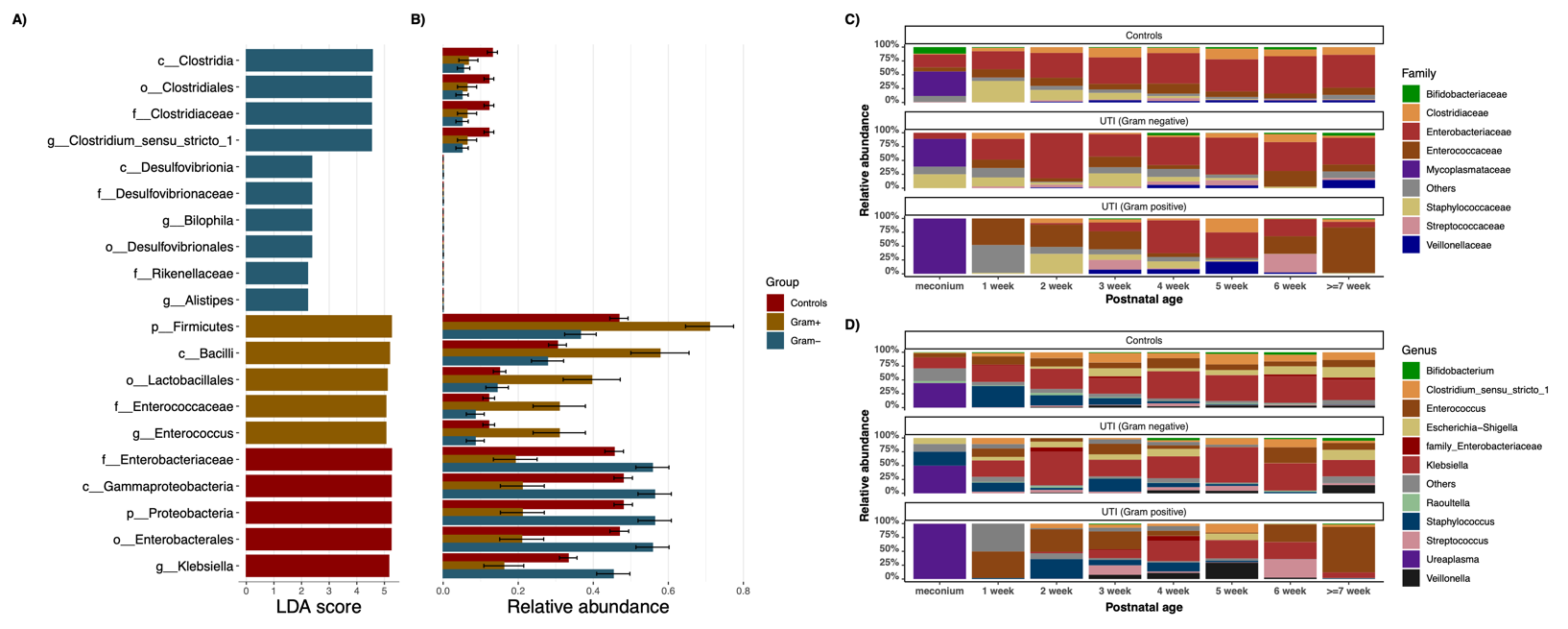


**(A-B)** Linear discriminant analysis and distribution of the relative abundance of distinct bacterial taxa that were significantly enriched in each group, respectively. **(C-D)** Disrupted gut microbiome development trajectory at the family level **(C)** and at the genus level **(D)** in stools of UTI infants.

**Figure S2. Increased abundance of pathogen-associated taxa in stools of UTI infants**.


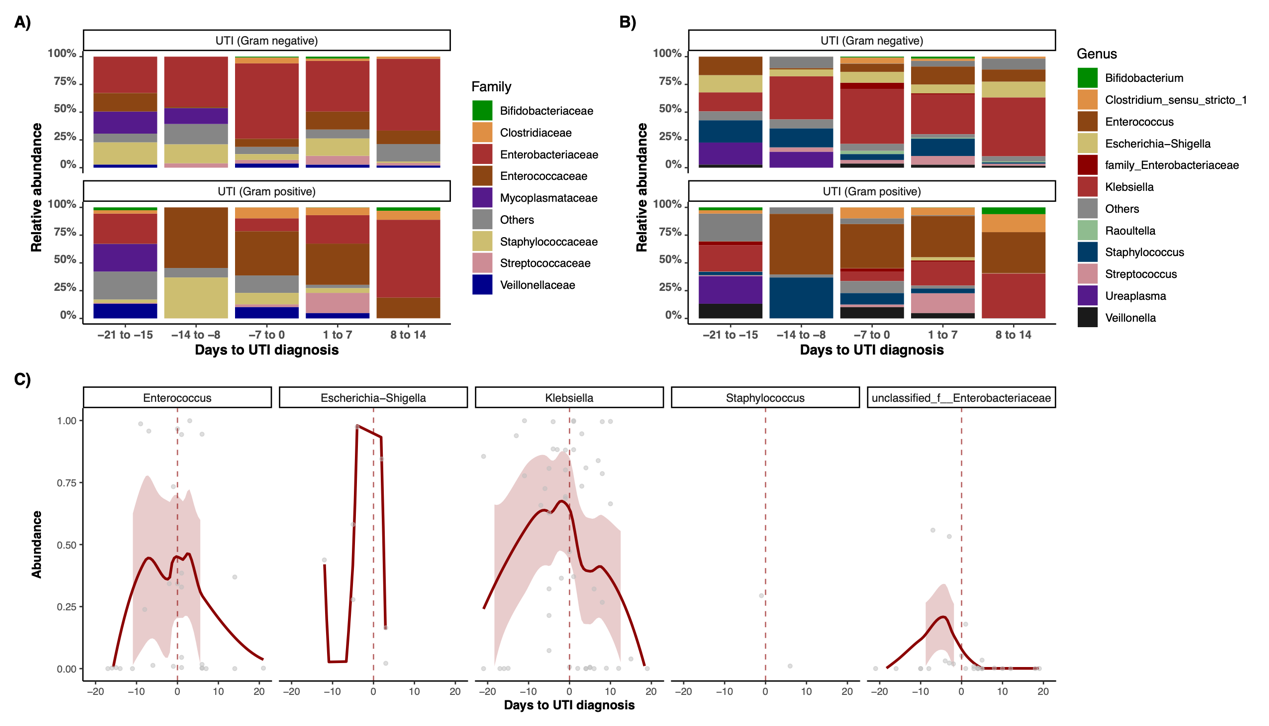


**(A-B)** Longitudinal change of gut microbial composition at the family level **(A)** and the genus level **(B)** according to the time of UTI onset. The distribution was according to the gram-staining classification of UTI pathogen. **(C)** Longitudinal change of relative abundance of pathogen-specific taxa at the genus level for different UTIs according to the time of UTI onset. Smoothed lines result from LOESS (locally estimated scatterplot smoothing) and indicate a longitudinal change of all data.

**Figure S3. Distinct pathogen-specific gut microbiome profiles in UTI infants prior to infection onset.**


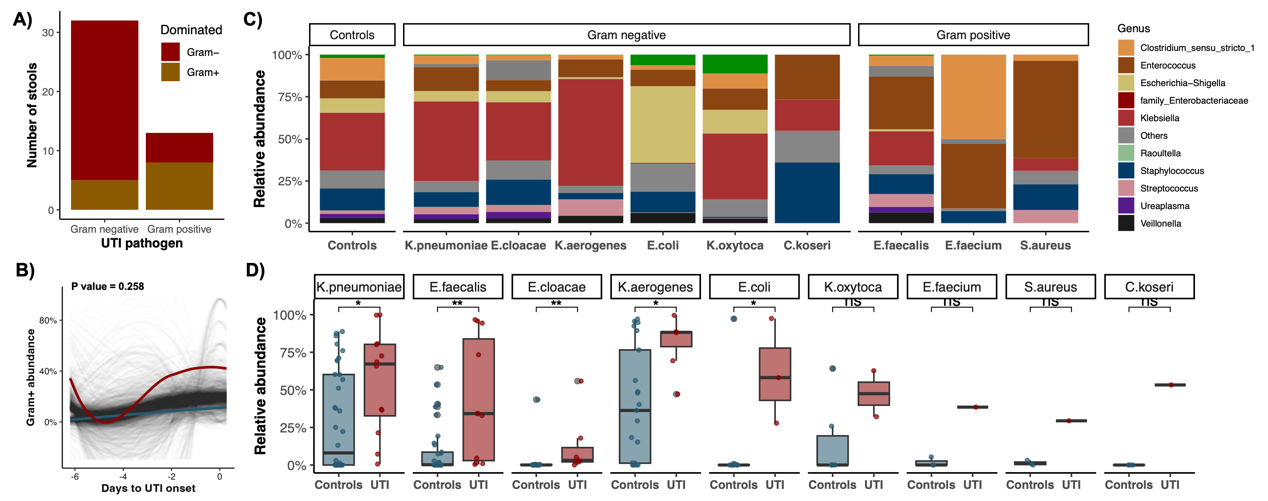


**(A)** Distribution of dominant taxa at Gram-staining level of pre-UTI samples of infants with Gram-negative UTI and Gram-positive, respectively. **(B)** Longitudinal changes in pathogen-specific abundance at genus level between matched samples of UTI infants with Gram-positive pathogens and non-UTI infants, using SplinectomeR with permuted spline test (permutations = 999). **(C)** Microbial composition at the genus level of pre-UTI samples of UTI patients with different pathogens and matched controls. **(D)** Boxplot revealed the difference in microbial abundance of matched samples between UTI infants and controls at the genus level, respectively.

**Figure S4. Volcano plot revealed the differential abundance of virulence factors of Escherichia coli UTI patients and matched controls (n = 12).**

**
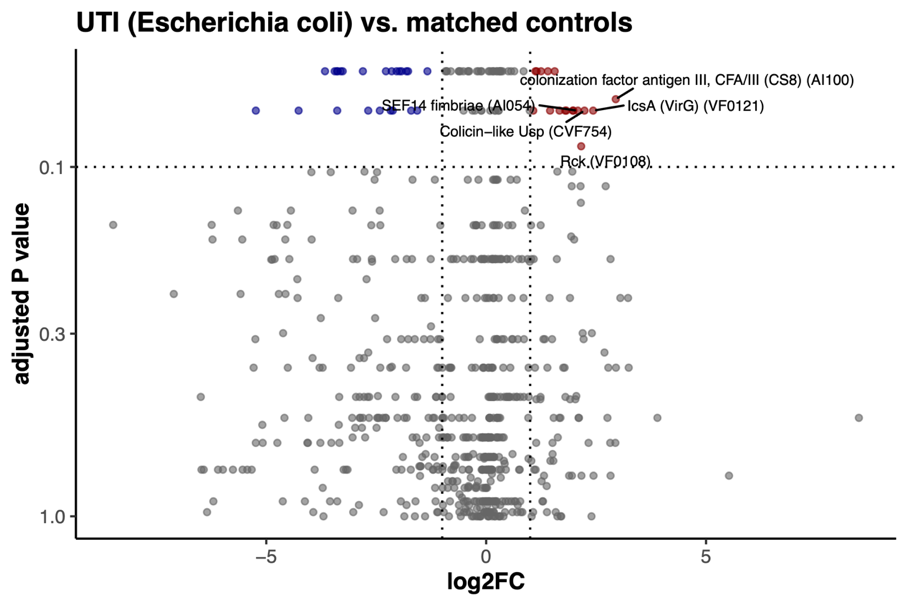
**

**Figure S5. Change of calprotectin level in matched samples of UTI infants and controls.**


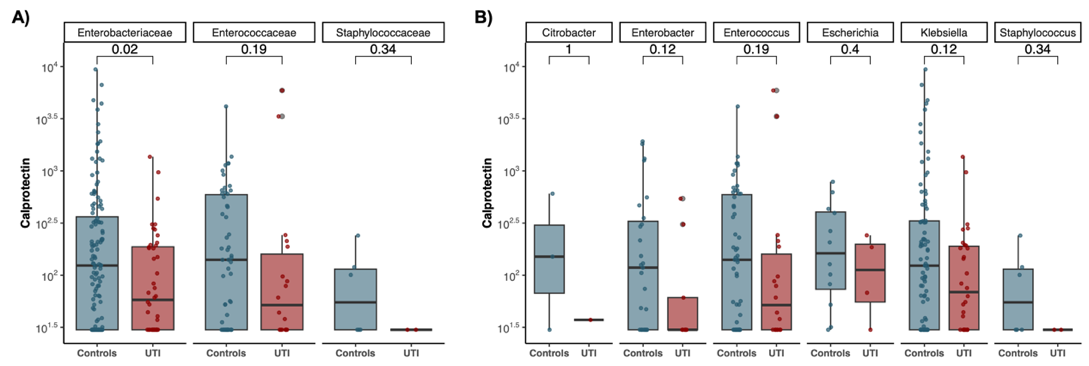


Boxplot revealed the difference in FC levels of matched samples between controls and UTI patients according to their pathogens at the family level **(A)** and the genus level **(B)**, respectively.
